# Ecotoxicological Effects of Aflatoxins on Earthworms under Different Temperature and Moisture Conditions

**DOI:** 10.3390/toxins14020075

**Published:** 2022-01-21

**Authors:** Tanya Fouché, Sarina Claassens, Mark Steve Maboeta

**Affiliations:** 1 Department of Environmental Science, University of South Africa, Private Bag X6, Florida 1710, South Africa; 2 Unit for Environmental Sciences and Management, North-West University, Private Bag X6001, Potchefstroom 2520, South Africa; sarina.claassens@nwu.ac.za (S.C.); mark.maboeta@nwu.ac.za (M.S.M.)

**Keywords:** aflatoxins, earthworms, soil ecotoxicology, soil moisture, temperature, climate change

## Abstract

Aflatoxin contamination remains one of the most important threats to food safety and human health. Aflatoxins are mainly found in soil, decaying plant material and food storage systems and are particularly abundant during drought stress. Regulations suggest the disposal of aflatoxin-contaminated crops by incorporation into the soil for natural degradation. However, the fate and consequences of aflatoxin in soil and on soil organisms providing essential ecological services remain unclear and could potentially pose a risk to soil health and productivity. The protection of soil biodiversity and ecosystem services are essential for the success of the declared United Nations Decade on Ecosystem Restoration. The focus of this study was to investigate the toxicological consequences of aflatoxins to earthworms’ survival, growth, reproduction and genotoxicity under different temperature and moisture conditions. Results indicated an insignificant effect of aflatoxin concentrations between 10 and 100 µg/kg on the survival, growth and reproduction but indicated a concentration-dependent increase in DNA damage at standard testing conditions. However, the interaction of the toxin with different environmental conditions, particularly low moisture, resulted in significantly reduced reproduction rates and increased DNA damage in earthworms.

## 1. Introduction

Fungal toxins (mycotoxins) are often toxic to plants, animals and humans and are a common threat to food safety. Of the more than 400 types of mycotoxins, aflatoxins are considered to be the most toxic and carcinogenic. Exposure to aflatoxin B_1_ (AFB_1_) poses a significant health risk for humans [1,2] and other living organisms, including plants [3,4], mammals [5], birds [6], insects [7,8,9] and fish [10]. Most countries in the world regulate aflatoxin concentrations in food and feed products. When aflatoxins reach concentrations exceeding the accepted levels, regulations suggest that contaminated food products are discarded by burning or working the material back into the soil for natural degradation [11]. When contaminated crops are worked into the soil, it increases natural concentrations and prolongs the duration of contamination due to the gradual release of the toxin [12]. Moreover, increased concentrations can alter the ecological balance, potentially posing a risk to soil health.

So far, only a few studies have investigated the consequences of aflatoxins for soil organisms [12]. The exposure of soil organisms to toxins in the soil is influenced by various mechanisms such as adsorption and release from the soil binding sites, interactions with the soil microbial community and the metabolic transformations of the toxin in the soil solution [13], as well as the environmental conditions under which it occurs. Studies from the early 1980s found that AFB_1_ does not persist in the soil for long as soil microbes degrade it into less toxic metabolites (AFB_2_ and AFG_1_ and AFG_2_) in a relatively short period. Later studies found that AFB_1_ may persist in soil for up to 120 days [14] when adsorbed to the soil binding sites. Once bound to the soil binding sites, AFB_1_ is mostly resistant to microbial degradation [14]. The effect of AFB_1_ on the growth, reproduction and DNA damage in the soil nematode (*Caenorhabditis elegans)* were investigated via a filter paper contact test [8]. Results indicated toxin-induced DNA damage, germline cell death and significant inhibition of growth and reproduction at concentrations between 30 and 100 µg/L [8,15]. Harmful effects on earthworms (*Eisenia fetida*) exposed to AFB_1_ via the filter paper contact test were observed related to their physical fitness and behaviour, including excessive mucus secretion, sluggish movement, coiling and swelling of the reproductive organs [16]. These studies used filter paper tests and did not include exposure in soil media. Schrader et al. [17] demonstrated that the mycotoxin, deoxynivalenol (DON), is incorporated into earthworm gut and body wall tissue after feeding on the fungi-infested crop residues in a soil medium. Although the DON concentrations in the earthworm gut declined over time, the possible toxic effects on the earthworm demographic processes (growth and reproduction) were not established. Similarly, the consequences of AFB_1_ and its degradation products for soil organisms [12] and soil biodiversity [16] remain unclear [18].

The loss of soil biodiversity has become a serious issue for global soil quality, especially in arable soils under intensive agriculture. Soil environments are complex systems considered the main reservoir of global biodiversity [19] and include diverse soil communities consisting of micro-and macro-organisms, e.g., bacteria, fungi, nematodes, mites, enchytraeids, springtails, ants, beetles and earthworms. The World Soil Charter recognises the critical importance of soil biodiversity for supporting soil functions and, therefore, providing, regulating and maintaining a diverse range of ecosystem services [20]. Ecosystem services refer to the subset of processes provided by an environmental compartment. The value of protecting soil biodiversity and ecosystem services to meet various sustainable development goals (as proposed by the United Nations) is widely acknowledged [19,21]. Economic growth and human well-being, therefore, depend on healthy soil. Unfortunately, the Status of the World’s Soils Resources report [20] concluded that most of the world’s soil resources are in poor or very poor condition, and urgent action is required, especially in developing countries where people are more vulnerable. The protection of soil biodiversity has also become essential for the success of the declared United Nations Decade on Ecosystem Restoration (2021–2030) [19].

Earthworms play an ecologically significant role in the soil ecosystem. They are considered ecosystem service mediators [22] due to their significant contribution to soil’s physical, chemical and biological processes. The activity of earthworms affects many essential soil processes, including the soil organic matter and nutrient dynamics and the activity of many other essential soil macro- and micro-fauna [22,23] and flora [24]. They are also essential biological regulators of plant pathogens such as fungi [25]. By regulating the fungal population, earthworms may regulate some of the harmful toxins associated with fungal populations, thus reducing the risk of environmental pollution as an ecosystem service [26]. They are resilient, widespread and have relatively uniform characteristics that classify them as good bioindicators of soil health [27]. They have been used extensively in ecotoxicological studies due to their ability to reflect trends in other species and their sensitivity to even the slightest changes in their environment [28,29].

According to the Intergovernmental Panel on Climate Change report (IPCC) [30], it is very likely that the variability in natural climates will continue to impact terrestrial ecosystems in the future. Extreme climatic events over the past decade, such as floods and extreme drought conditions, have resulted in more sudden and severe changes in soil temperature and moisture conditions rather than gradual shifts [20]. Several studies have indicated that climate change might influence mycotoxin production [31], making it a more considerable risk in the future. It is suggested that climate change significantly impacts the stages and rates of toxigenic fungi development and toxin production [32], which might modify host-resistance and host–pathogen interactions. A study by Sanders et al. [33] found a relationship between soil moisture, temperatures, the percentage of peanut plants colonised by *Aspergillus* spp. and the total aflatoxin concentrations on the plant material. In irrigated soil, aflatoxin could not be detected; however, in cooler, drought soil, total aflatoxin concentrations ranged between 0 and 19 µg/kg for edible plant crops and between 66 and 2553 µg/kg in oil crops. In drought-heated soil, the total aflatoxin concentrations range of contaminated plants increased to 417–10,516 µg/kg. Similar findings report increased aflatoxin contamination in groundnut [34] and corn [35] after prolonged drought conditions. The increased concentrations during drought conditions highlight the necessity to better understand how climate change may influence the risk of pre-and postharvest aflatoxins in the soil environment.

Risk assessments of toxic substances historically relied only on whole-organism endpoints directly related to demographic processes such as survival, growth and reproduction [36]. While organisms can show some tolerance towards toxicants, especially at lower concentrations, it does not imply that there are no effects, as there could be some physiological changes, even in the absence of mortality [37]. Complementary investigations that incorporate both demographic and mechanistic aspects of the biological effects of toxic substances, sometimes overlooked in ecological risk assessments, have developed over the past 20 years [28]. Mechanistic aspects include the biochemical and molecular basis by which the toxin exerts an effect; for example, the consequences of exposure on metabolism can be evaluated by its genotoxicity. The comet assay or single-cell gel electrophoresis assay is a sensitive biomarker to identify and quantify genotoxicity. It can examine the double-strand breaks of DNA in any individual eukaryotic cell and has been successfully applied as a monitoring tool to detect DNA damage in humans [38], other mammals [39], plants, freshwater organisms [40] and invertebrates [41].

Risk assessments applied in laboratory tests are generally based on standard temperature and moisture conditions for reproducibility and comparison between different studies [42]. The Organisation for Economic Co-operation and Development (OECD) provides standard temperatures (20 ± 2 °C) and often moisture conditions when testing chemicals in soil organisms [43]. However, existing monitoring and assessment methods may no longer be robust enough to detect adverse changes in organisms after exposure [44]. Changing climate conditions can alter the toxicokinetics of toxic substances [36,45]. Several studies report the impact of different temperature regimes on the toxicity of agricultural pesticides [46,47,48,49] and metals [50] to soil organisms. These impacts vary as soil organisms respond differently to different toxins and chemicals under different temperature conditions. Garcia [46] assessed the impact of temperature on the toxicity of two fungicides and an insecticide on two different invertebrate species (earthworms and isopods). Lower toxicity was indicated for the fungicides at higher temperatures (28 °C) but higher toxicity for the insecticides. Bandow et al. [51] reported increased susceptibility of Collembola to the fungicide pyrimethanil at 26 °C compared to 20 °C. Increased toxicity of several common pesticides has been reported under increased temperatures (25 °C) [48]. Similarly, increased toxicity of three common agricultural pesticides (chlorpyrifos, dimethoate and deltamethrin) were observed under tropical temperatures (26–28 °C), even if the concentrations were not considered a risk in their study [49]. Further, temperature-induced variations in earthworm enzymatic activities and proteins that may contribute to compensatory changes at the cellular metabolic level have also been reported [52].

Temperature is generally not the only environmental factor that plays a role. In most cases, increased temperature is also associated with other soil factors such as moisture conditions. Most studies primarily focus on different temperatures and do not always consider a combination of temperature and moisture conditions. The bioaccumulation and toxicity of metals in earthworms and enchytraeids under different climate change scenarios were investigated [45,50] Findings suggest that different air temperature and soil moisture combinations affect metal bioaccumulation kinetics in these organisms. Hackenberger et al. [29] found that different temperature and moisture combinations affected earthworm enzyme activity and the organism’s behavioural response after exposure to agricultural pesticides. Low moisture and high temperature in soil have been reported to increase earthworms’ physiological stress, resulting in decreased protein synthesis and tissue protein levels [53]. The temperature and moisture-induced decrease in protein synthesis might affect DNA repair activities in organisms [54]. It has, therefore, become necessary to monitor and assess the effects of different toxic substances under a broader range of environmental conditions. If climate conditions continue to change, it could potentially increase the risks of aflatoxin contamination in soil ecosystems in the future [32].

To address some of these knowledge gaps, the current study aimed to investigate the toxicological consequences of aflatoxins to earthworms (*E. andrei*) under different temperature and moisture conditions. The specific objectives of the study were:

- Assess whether aflatoxin affects earthworms’ demographic processes (survival, growth and reproduction) using a standard OECD test.
- Assess the genotoxicity of aflatoxin to earthworms using the comet assay.
- Assess whether different temperatures (21 °C and 26 °C) and soil moisture conditions (30% and 50% of soil water holding capacity) affect the toxicity of aflatoxins to earthworms.

## 2. Results

### 2.1. Aflatoxin Concentrations in the Soil

**Figure 1:**
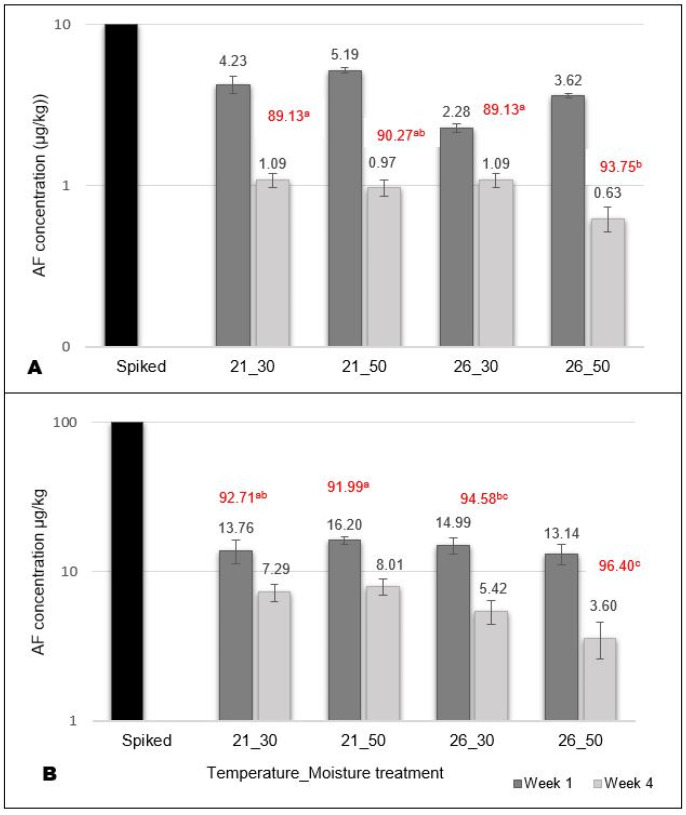
Mean aflatoxin concentrations (AF) detected in soil 72 h (Week 1) and 32 days (Week 4) after spiking the soil. (**A**) Soil spiked with 10 µg/kg, and (**B**) soil spiked with 100 µg/kg. Percentage (%) decrease from the initial spiked amount is indicated in red. Different alphabetical letters (a, b and c) indicate a significantly (*p* < 0.05) different decrease (%) in detected concentrations.

A total aflatoxin ELISA kit was used to detect and quantify the AFB_1_ and its breakdown products (AFB_2_ and AFG_1_ and AFG_2_). Low available concentrations were detected in the OECD soil (Figure 1) but confirmed that aflatoxin was present in the soil for the study duration. Higher concentrations were generally detected at 21 °C than 26 °C for both concentration treatments (10 µg/kg and 100 µg/kg) in week 4 (Figure 1). The percentage (%) decrease in detected concentration levels over four weeks was lower in the drier (30% WHC) soil, suggesting that the aflatoxin concentration degraded more slowly in dry soil. The degradation potential (% decrease) was significantly (*p* < 0.05) higher at increased temperatures and moisture conditions.

### 2.2. Earthworm Survival, Weight Change and Reproduction

The aflatoxin concentrations used in this experiment were at sublethal doses for earthworms. The LD_50_ of AFB_1_ to earthworms as 168.5 µg/mL have been determined during a contact paper test [16]. All control treatments had a survival rate above 90%, which met the validity criteria of the OECD 222 [43].

There was no significant (*p* > 0.05) difference in the survival rate between treatments at the same temperature, but the survival was lower at 26 °C than at 21 °C The percentage change in the earthworms’ mean body weight was noted as a measure of their growth. Results for the average weight change indicated a decrease in the mean body weight of less than 10% in all the groups. The earthworms were cultured in a different substrate with a higher percentage of available food. The weight loss percentage of earthworms after 4 weeks in the aflatoxin-treated soil was generally less than the control groups in each environmental group (same temperature and moisture conditions); however, the analysis of variance (ANOVA) found no statistical difference (*p* = 0.06) between the control and aflatoxin treatments.

**Figure 2:**
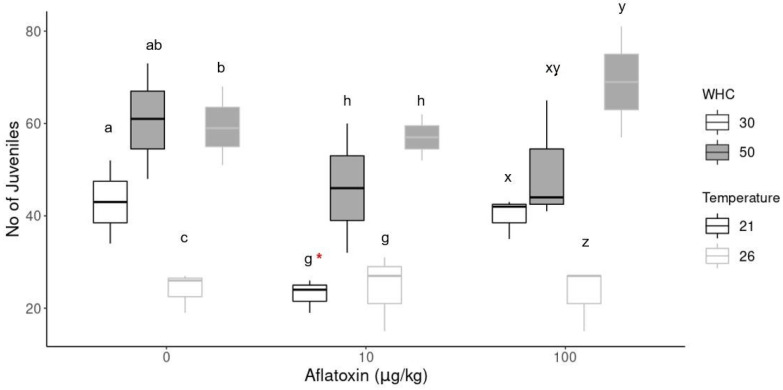
Reproductive output of earthworms. The * indicates a significant difference (*p* < 0.05) between concentration treatments at the same environmental conditions. Different alphabetical letters (a, b, c in 0 µg/kg; g, h in 10 µg/kg; x, y, z in 100 µg/kg) indicate significant differences (*p* < 0.05) within the same concentration group.

Figure 2 shows the difference in the reproduction of earthworms at different aflatoxin concentrations and different environmental conditions. The number of juveniles at low aflatoxin concentrations (10 µg/kg) at 21 °C with 30% WHC was significantly (*p* = 0.03) less than its control sample (indicated by *). There was no difference in the number of juveniles at any other aflatoxin treatment compared to its control sample at the same environmental conditions. However, there were significant differences in the number of juveniles hatched at the same concentration under different environmental conditions.

Soil temperature and moisture are key factors that influence earthworm growth, survival, reproduction [55] and other life cycle traits such as weight and cocoon incubation time. Drier soil conditions (30% WHC) decreased the number of juveniles hatched, especially at increased temperatures (26 °C). Conversely, increased soil moisture (50% WCH) increased the number of juveniles hatched. In the aflatoxin treatment groups, the biggest difference was observed at the increased temperatures (26 °C) because the least number of juveniles hatched in the treatments with 30% WHC. In contrast, the highest number of juveniles were produced in the 50% WHC group, but the adults also had the highest weight loss percentage in this treatment. In the control group (0 µg/kg), the highest number of juveniles were produced at standard temperatures (21 °C) with 50% WHC.

### 2.3. Genotoxicity-Comet Assay Results

**Figure 3:**
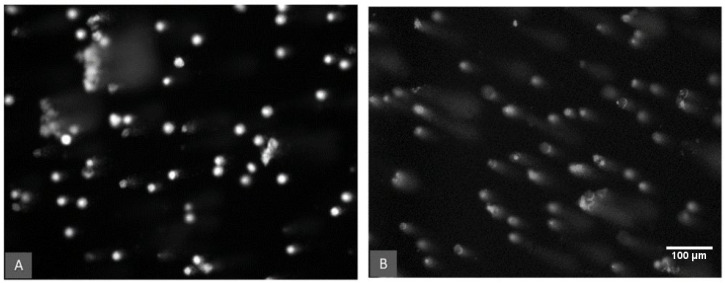
DNA damage of earthworm coelomic cells as viewed under a fluorescence microscope. Comparison of the aflatoxin 100 µg/kg treatment at 21 °C. (**A**) Cells at 50% water holding capacity treatment. (**B**) Cells in the 30% water holding capacity treatment and a significantly (*p* < 0.001) higher percentage of cells with DNA damage represented by the characteristic comet tails.

**Figure 4:**
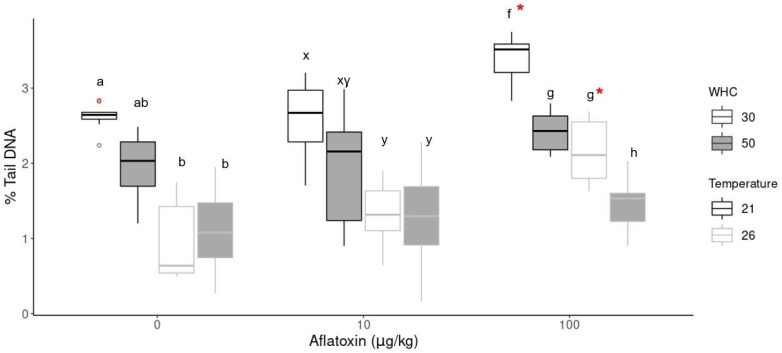
DNA damage in earthworm cells as % tail DNA at different aflatoxin concentrations (0, 10 and 100 µg/kg), temperature and moisture treatments. An * indicates a significant difference (*p* < 0.05) between the aflatoxin concentration and its control treatment at the same moisture and temperature conditions. Significant differences (*p* < 0.05) within the same concentration groups are indicated by different alphabetical letters (a, b in 0 µg/kg; x, y in 10 µg/kg; f, g, h in 100 µg/kg).

Figure 3 shows an example of the observed DNA damage in earthworm coelomic cells measured as the tail intensity of the DNA strand breaks (% tail DNA) for the aflatoxin 100 µg/kg treatment group. The comet assay data were compared based on the aflatoxin concentration, the temperature and the moisture treatments. DNA damage in the control samples (0 µg/kg) is assumed to be the background values derived from endogenous and natural exogenous sources [54]. These background levels differed significantly (*p* < 0.05) between the two temperature treatments (Figure 4). Control earthworms had significantly lower DNA damage at 26 °C compared to 21 °C.

In the aflatoxin treatments, the analysis of variance (ANOVA) indicated significant differences between the concentration groups at the same environmental conditions. Tukey’s HSD indicated significantly (*p* < 0.05) higher DNA damage in the highest concentration group (100 µg/kg) with 30% WHC compared to the control (0 µg/kg) and the 10 µg/kg groups (indicated with * in Figure 4). The aflatoxin treatment group at 21 °C was significantly higher (*p* < 0.001) than all other treatments. The DNA damage of the 100 µg/kg group with 50% WHC was slightly increased at both temperatures but not statistically significant compared to its control group. These results indicate genotoxicity at the increased concentration group under drought conditions. There was also no statistical difference between the control and 10 µg/kg groups.

The comet assay results were further compared at the same concentration with different temperatures and soil moistures (Figure 4). The same trend was observed in the 10 µg/kg and 100 µg/kg groups. Increased soil temperatures (26 °C) resulted in less DNA damage of earthworm coelomic cells than standard temperature (21 °C). The lower DNA damage at 26 °C correlates with the lower detected aflatoxin concentrations at 26 °C compared to 21 °C (Figure 1). The lowest DNA damage was observed in earthworms kept at 26 °C and 50% WHC. In contrast, the highest level of DNA damage was observed in earthworms at standard temperatures (21 °C) with drier soil conditions (30% WHC), irrespective of the aflatoxin concentration.

### 2.4. Interaction Effect of Climate Conditions on the Toxicity of Aflatoxin

Three-way ANOVA assessed the interaction effect of the aflatoxin concentration, soil moisture (WHC) and temperature on reproduction and DNA damage (Table 1). The study demonstrated that different environmental conditions might affect the toxicity of aflatoxin in soil. Results indicated a statistically significant (*p* < 0.001) interaction effect between moisture vs. temperature on reproduction and DNA damage. There was also a significant interactive effect of the aflatoxin concentration vs. moisture in the genotoxic study. Post-hoc analysis shows the specific combinations of moisture and temperature that resulted in an interaction effect (Table 2). There was no statistical interaction effect of soil moisture and temperature on weight loss. The same moisture levels at different temperature combinations did not prove significant for the reproduction test. However, there was a significant (*p* < 0.05) interaction effect between moisture and temperature in the genotoxicity study. The only combination that did not have a significant interaction effect was different moisture levels at the increased temperature.

## 3. Discussion

Aflatoxin AFB_1_ has a relatively short half-life (≤5 days at 28 °C) in soil [12] and degrades quickly into other metabolites (AFB_2_ and AFG_1_ and AFG_2_). However, soil with higher organic matter and clay content form an aflatoxin conjugate with the soil-binding sites resistant to microbial degradation and may result in the AFB_1_ persisting in the soil for much longer [14]. ELISA is a quick and reliable technique to detect and quantify aflatoxins. These assays are mostly produced for detection in food and feed products. The recovery rates for soil matrices using ELISA are very low because organic solvents, such as chloroform or methanol used in this study, seldom extract the toxin bound to the soil binding sites effectively [3]. However, for this study, the ELISA results sufficiently quantified available aflatoxin concentrations and indicated low aflatoxin levels still present in the treated soil four weeks after spiking. These low concentrations mostly represent the free aflatoxin in the soil. *Eisenia andrei* are primarily detritivorous organisms that prefer to feed on organic matter such as decaying plant material. However, they also exhibit geophagous feeding traits [56], suggesting that the earthworms could have been exposed to the soil’s free aflatoxin concentrations and the bound aflatoxin.

The ELISA results showed that different temperature and moisture combinations affected the concentration decrease over four weeks. The slower concentration decrease % in the drier soil suggested that the toxin persisted longer in the drier soil compared to the wetter soil. The natural degradation of aflatoxin under different temperature and pH values have been investigated [57], but little information is available about the effects of moisture conditions on the natural degradation of aflatoxin. It is, however, known that the sorption of AFB_1_ onto the soil particles is reduced in pre-saturated soil [58], suggesting that more AFB_1_ will be available for microbial degradation under moist conditions, whereas in dry conditions, the AFB_1_ will become less available for microbial degradation.

Growth is considered an important measure of an individuals’ fitness after exposure to a chemical or toxin [59]. The environmentally relevant aflatoxin concentrations used in this study did not significantly affect earthworm survival, growth and reproduction. Higher concentrations may, however, still result in harmful effects on earthworms. A concentration-dependent decrease in reproduction and a 40–60% size reduction were reported in nematodes (*C. elegans*) after aflatoxin exposure of as low as 3 µM [15]. Degenerative changes have been reported in the reproductive areas of earthworms exposed to levels between 150 and 400 µg/L [16]. According to Sing et al. [60], increased temperatures typically increase earthworm abundance and may accelerate earthworm growth, whereas extreme climates such as drought and flooding might have more deleterious effects. The environmental conditions had a more pronounced effect on the earthworm survival, growth and reproduction than the aflatoxin. Increased temperatures generally resulted in a higher weight loss percentage, decreased survival and increased reproduction under standard moisture conditions, although it was not statistically significant compared to 21 °C. Soil moisture did not affect weight loss but significantly affected reproduction. Even in the absence of the toxin, the significantly reduced reproduction rates at higher temperatures and decreased moisture indicate the physiological stress [53] for the earthworms at these conditions. Similar body weight changes in *E. andrei* under various climate scenarios were reported [47,50]. Lima et al. [47] found a synergistic effect between carbaryl toxicity and soil moisture on survival and weight loss in earthworms, whereas González-Alcaraz and van Gestel [50] reported increased weight loss of control earthworms kept at 25 °C compared to 20 °C with no effect by soil moisture. In contrast, Diehl and Williams in [60] found decreasing body weight due to lowered soil moisture in *E. fetida*. There was evidence of a trade-off between earthworm reproduction and growth that may affect their response to toxicants [28]. In the aflatoxin treatments, the groups with the highest weight loss (26 °C and 50% WHC) also had the highest number of hatched juveniles, suggesting a different pattern of individual resource expenditure. The group with the lowest weight loss (10 µg/kg at 21 °C with 30% WHC) resulted in a significant decrease in the number of juveniles. The slower concentration decrease in the drier soil (Figure 1) could also have contributed to this.

This size difference observed in the juveniles from the different moisture treatments suggests increased moisture-delayed cocoon hatching. Asynchronous and delayed hatching and the ability of the cocoons to remain viable for extended periods under favourable environmental conditions allow them to maximise their reproductive output [61]. In this study, increased moisture prolonged the cocoon incubation period. In contrast, drier soil conditions decreased the cocoon incubation period, suggesting that the juveniles hatched earlier, which is why they were bigger at 10 weeks. Optimal temperatures for most earthworm cocoons have been reported as 15 °C and 24 h darkness, and increased temperatures may increase the cocoon incubation period [61]. This study found that soil moisture had a more pronounced effect on the cocoon incubation period than the temperature or the aflatoxin treatments.

Once AFB_1_ is metabolised, it forms a genotoxic metabolic intermediate, AFBO, that can bind to DNA to form an aflatoxin-DNA adduct or induce DNA damage [8]. Increased levels of DNA damage were observed in earthworm coelomocytes of the 100 µg/kg group, indicating the possible genotoxicity of aflatoxin in the soil at these concentrations. The groups with the highest DNA damage correlated with the higher detected aflatoxin concentrations in the soil. The genotoxicity of aflatoxin to soil nematodes (*C. elegans*) was indicated after exposure to concentrations between 30 and 100 µM (9–32 ppm) at optimal temperatures (15–20 °C) [15], although the study was not conducted in a soil medium, and soil moisture was not a determining factor.

As poikilothermic organisms, earthworms generally have increased metabolic activity at increased temperatures up to a threshold level [50]. The results indicated lower DNA damage at increased temperatures in both the control and toxin groups. Although the higher temperature is considered sub-optimal for *Eisenisa* species [55], they can respond to temperature changes up to 28 °C by adjusting their enzymes capacities [52]. Tripathi et al. [52] investigated the temperature-dependent changes in metabolic enzymes and proteins in earthworms ranging from 12–44 °C and found that increased temperatures up to 28 °C decreased the activity of enzymes involved in energy production but contributed to compensatory changes in enzymes involved in the cellular metabolism, such as increased protein synthesis and possibly gene expression [52]. DNA repair mechanisms in earthworms are facilitated by enzymes, although the exact mechanisms are unknown [54]. Studies have shown that the DNA repair can be very rapid, with strand breaks being repaired with a half time of less than thirty minutes and as short as three minutes [39]. The lower DNA damage at increased temperatures is possibly due to the increased metabolic activity and compensatory enzymatic changes in the earthworm metabolism. These results are consistent with the findings of another study [46] that found a temperature-dependent decrease in the toxicity of fungicides under tropical conditions (28 °C).

In contrast, elevated DNA damage was observed at the increased temperature in combination with low moisture and high aflatoxin concentrations (100 µg/kg with 30% WHC). Although soil moisture is a known stressor to earthworms [60], the effect of one environmental factor cannot be interpreted on its own because the interaction of more than one environmental factor with the chemical stressor might increase the toxicity of these chemicals for organisms [36,62]. Further, external factors such as temperature, moisture and the accumulation of reactive oxygen species (ROS) due to a chemical stressor may also impact the enzymes involved in metabolic detoxification [63]. The significantly (*p* < 0.05) higher levels of DNA damage of the 100 µg/kg at increased temperatures with low moisture indicated the interaction of the toxin with the environmental conditions. Similarly, other studies reported enhanced metal detoxification under increased temperature (25 °C) with 50% moisture but significantly lower metal detoxification at the same temperature with low (30%) moisture [45,50] and suggested that the combination of the warmer and drier environment could have hindered the earthworm metabolic performance. In many studies, the interaction of only one environmental factor with a chemical stressor is considered; however, the interaction of more than one environmental factor with the chemical stressor is more realistic [36] as temperature and moisture are inherently linked in the soil. A significant three-way interaction effect of soil type vs. moisture content vs. temperature was observed on the earthworm growth [62]. Hackenberger et al. [29] found a statistically significant three-way interaction effect of pesticide concentration, temperature and moisture that affected earthworm enzyme activity and their response to pesticides. There was no evidence of a three-way interaction effect between concentration levels, temperature and moisture in this study. However, the possibility at higher concentrations should not be excluded because the moisture and temperature interaction was indicated in both the reproduction and genotoxicity tests. High levels of DNA damage can lead to genome disturbances that may impair growth, reproduction and population dynamics in the long term [63]. The fact that the DNA damage was still elevated after 30 days of exposure suggests the possibility that the toxin may cause more permanent damage during drought conditions.

The results demonstrate the sensitivity of the comet assay to determine the effect of different environmental conditions (temperature and soil moisture) on the genomic functioning of the earthworm. The increased levels of DNA damage detected in uncontaminated soil at low soil moisture may have consequences for cell functioning and how organisms deal with other stressors (toxins) in their environment [63]. The genotoxic biomarker proved to be more sensitive when evaluating the toxicity of aflatoxin in the soil than whole-organism responses such as survival, growth and reproduction. Smit et al. [64] found DNA damage to be 35–50 times more sensitive for evaluating oil toxicity in marine species than whole-organism responses. However, DNA damage alone can only relay information about the individual and must be complemented with growth and reproduction responses to predict possible effects at the population level [63]. Complementary investigations on the reproductive output and the genotoxicity in earthworms in this study suggest that aflatoxin might be harmful at the population level during climate change.

## 4. Conclusions

Results indicated an insignificant effect of aflatoxin concentrations between 10 and 100 µg/kg on the earthworms’ (*E. andrei*) survival, growth, and reproduction in an OECD soil medium. The presence of the toxin reduced the number of juveniles but also prevented the same level of weight loss compared to the control groups, although it was not statistically significant. Comet assay results indicated a concentration-dependent increase in DNA damage after 30 days of exposure to aflatoxin, suggesting that increasing aflatoxin concentrations might influence the health of soil organisms.

Different combinations of temperature and soil moisture conditions resulted in different effects. Increased temperatures generally resulted in lower survival rates and increased weight loss but increased reproductive output and showed less DNA damage, indicating their ability to adapt a different pattern of individual resource use. Moisture had a more pronounced effect on the population performance in terms of reproduction and DNA damage. Significantly reduced reproduction rates at higher temperatures and decreased moisture, even in the absence of the toxin, indicate the physiological stress for the earthworms at these conditions; however, further investigations using different biomarkers conducted in natural soil are recommended to confirm these findings.

Although limited effects of the toxin were observed at standard testing conditions, the exposure–effect outcomes of aflatoxin might be influenced by climate change due to the interaction of the toxin with environmental conditions. In particular, decreased moisture treatments resulted in a significantly decreased reproductive output at low aflatoxin concentrations and significantly more DNA damage with increasing aflatoxin concentrations. Complementary investigations on the reproductive output and the genotoxicity in earthworms suggest that aflatoxin might be harmful at the population level during climate change. Future studies using contaminated agricultural soil will be valuable in predicting aflatoxins’ effect in the natural environment. When using OECD soil, some variables in natural ecosystems that can affect the toxins’ bioavailability might be excluded and can alter the results.

## 5. Materials and Methods

### 5.1. Experimental Design

A laboratory experiment was conducted at the North-West University, South Africa to assess the effect of AFB_1_ on the survival, growth, reproductive output and DNA health of earthworms. The dynamic nature of the soil environment makes it very difficult to interpret and measure the ecological functions of secondary metabolites in the soil (Karlovsky, 2008). Therefore, toxicological studies often use artificial soil prepared according to standard guidelines to overcome some of the complexity and heterogeneity of the soil environment. Although some variables of natural ecosystems are excluded, which can affect bioavailability, it has practical advantages. Using artificial soil manipulates essential parameters such as soil organic matter (SOM) and the variability in adsorptive properties of different soil types. Artificial soil was prepared ten days before starting the experiment according to the standard guidelines set out by the Organisation for Economic Co-operation and Development [43] and used in all the treatment exposures. The artificial OECD soil consisted of (based on dry weight):

- 10% sphagnum-peat (Mystics).
- 69% quartz sand with a grain size between 50 and 200 μm.
- 20% kaolinite clay (obtained from Atlas Clay Group in Potchefstroom, South Africa).
- Chemically pure calcium carbonate (<1%) to obtain a pH of 6.5–7.

The water holding capacity (WHC) of the OECD soil was determined using a Sartorius moisture analyser. The mean WHC (100%) was determined from four replicate samples, and the 30% and 50% WHC values were calculated.

For this study, modifications were made in terms of the standard OECD guidelines for air temperature and soil moisture to represent a range of temperature and moisture conditions to assess if changing climate conditions might affect the toxicity of aflatoxins to earthworms. Four combinations of air temperature and soil moisture (as soil water holding capacity) were used and based on previous studies by other researchers [29,45]. Although the higher temperature is considered sub-optimal for *Eisenia*, it was included to represent soil temperature conditions encountered in a southern African context [65].

- 21 ± 1 °C + 50% (WHC)—standard temperature and moisture conditions prescribed by the OECD.
- 21 ± 1 °C + 30% (WHC)—standard temperature with drier soil conditions.
- 26 ± 1 °C + 50% (WHC)—increased temperatures with standard moisture conditions.
- 26 ± 1 °C + 30% (WHC)—increased temperatures with decreased soil moisture conditions.

### 5.2. Introduction of Aflatoxin into the Soil

Two different concentration treatments (10 and 100 µg/kg) were used for the study. The concentrations selected are comparable to actual concentrations found in environmental soil samples (Table 3).

Before introducing the toxin, the soil was moistened with dH_2_O to the desired water holding capacity (30% and 50%). Methanol was used as a solvent to prepare the liquid aliquots of the powdered AFB_1_ (Enzo Life Science, through Biocom Africa, Centurion, South Africa). Aflatoxins are typically introduced into the surface soil by infected plant material left to decompose [3,12]. The powdered aflatoxin was dissolved with methanol to obtain 10 µg/mL for the higher concentration treatment. Then, 1 mL of the 10 µg/mL aflatoxin was further diluted with methanol to obtain 1 µg/mL for the lower concentration treatment. For each vessel containing 600 g of soil, 6 mL of the aflatoxin solvent was mixed with 5 g dried horse manure containing sufficient quantities of wheat straw and placed on top of the moistened soil to introduce the desired aflatoxin concentration into the soil. The soil samples were weighed individually and placed under an extractor fume hood overnight in the dark at room temperature to allow the solvent to evaporate. After 12 h, the soil was weighed again, and any loss of weight was compensated for by replacing lost moisture with dH_2_O. The soils were left for another 12 h to stabilise before introducing the earthworms. Control soil samples (three replicates for each WHC) with 5 g of uncontaminated horse manure were moistened with dH_2_O to achieve the desired WHC of 30% and 50%, respectively, and left to stabilise for 12 h.

### 5.3. Aflatoxin Concentrations

Soil samples were analysed 72 h (week 1) and 32 days (week 4) after spiking with AFB_1_ to monitor and quantify bioavailable AFB_1_ concentrations during the experimental period. Aflatoxin concentrations were analysed using an indirect, competitive enzyme-linked immunosorbent assay (ELISA) according to the suppliers’ protocol (Elabscience total aflatoxin ELISA kit, E-TO-E006). The pre-treatment method for formula feed was most effective for extracting aflatoxin from the soil. The 96-well ELISA microtiter plate was prepared using 50 µL of the extracted solution. Each sample (*n* = 3) was inoculated in duplicate to ensure a consistent and accurate result. The optical density (OD) for each well was determined using a microplate reader at a wavelength of 450 nm. When optical density values were outside the detection limits, a further ten-times dilution was performed by mixing 0.5 mL of the supernatant and 0.5 mL of de-ionised water with 35% methanol to obtain a 100 times dilution. A standard curve was generated from the OD values of a range of six standard solutions (0–0.32 µg/mL). A four-parameter logistics (4PL) regression model was used to calculate the concentrations in the samples based on the standard curve.

### 5.4. Earthworm Survival, Weight and Reproduction Test

Lab-cultured adult earthworms (*Eisenia andrei*) were used for this study. *Eisenia* are amongst the most popular earthworm species found in compost and organic layers. Contaminated plant material introduces aflatoxin into the organic and compost layers and the soil [12], where these organisms are exposed.

Adult earthworms of reproductive age (visible clitellum) were acclimatised in clean experimental soil for 72 h. The earthworms were briefly rinsed, placed on absorbent paper and then weighed individually [43]. The mean starting weight of the worms was noted. Ten earthworms were introduced into each replicate vessel containing the soil. The weight of each vessel containing the soil, moisture at the desired WHC, 5 g of horse manure as food substrate (contaminated in aflatoxin treatments) and ten earthworms was noted and incubated in an environmental climate chamber at 21 ± 1 °C for 30 days. The experimental layout was duplicated in a second trial, using new soil and different earthworms, but the soil samples were incubated at 26 ± 1 °C.

The soil moisture level was maintained throughout the experimental period by weighing the vessels every seven days and adding dH_2_O to maintain the original water holding capacity percentage. In addition, 5 g of re-wetted horse manure was added weekly as a food source for the earthworms as per the guidelines set by the OECD [43] to ensure a normal cocoon production rate. After 30 days, the adult earthworms were removed from the vessels, counted (survival) and individually weighed after briefly rinsing and removing excess water. The average change in body weight as a percentage for the ten earthworms over the 30-day incubation period was determined for each vessel. Immediately after weighing the adult worms, three individuals per vessel (*n* = 9 per treatment) were placed on moist paper to extrude their gut content overnight for use in the comet assay. The vessels containing the cocoons and juvenile earthworms were incubated for an additional 30–40 days under the same conditions. Food (5 g of horse manure) was provided only once at the start of the second incubation period, and the moisture levels were monitored weekly. After the second incubation period, the number of cocoons and juveniles was collected and counted using a hand-sorting technique [43]. Each vessel was checked in triplicate (over three days) to remove all the juveniles and cocoons. There was very little evidence of cocoons or no cocoons in some samples, even though the juveniles were present. This was possibly due to the desiccation of the egg capsules at these conditions after the juveniles hatched.

### 5.5. Comet Assay

The single-cell gel electrophoresis assay (comet assay) was conducted 30 days after earthworm exposure according to the protocol used by Voua Otomo and Reinecke [67] to evaluate the extent of cellular DNA damage in earthworm coelomic cells. The OxiSelect™ Comet Assay kit (Cell Biolabs, Inc., San Diego, CA, USA) was used. All the comet assay solutions (lysis solution–pH 10, Mg and Ca free phosphate-buffered saline (PBS), alkaline solution and electrophoresis running solution-pH 13) were prepared according to the Cell Biolabs protocol a day before the start of the assay and refrigerated at 4 °C. Twelve hours after removing the adult worms from the soil, coelomic cells were harvested from three earthworms per sample (*n* = 9) using a non-invasive technique [41,67]. Harvested cells were washed three times, exposed to the lysing solution to enable denaturation and unwinding of the DNA, after which electrophoresis was conducted. Voltage was applied for 30 min at 1 volt/cm, and the volume of the electrophoresis solution was adjusted to produce a current of 300 mA. After electrophoresis, the slides were rinsed twice in cold distilled water and once in 90% ethanol for five minutes each, air-dried and stored in the dark until analysis.

The Comet IV software package was used to analyse the DNA damage in the coelomic cells. A minimum of 50 randomly selected cells per worm (*n* = 9 × 50 cells) were analysed under a fluorescence microscope after staining the slides with an intercalating staining agent. The percentage of DNA that migrated away from the nucleus, represented by the tail intensity parameter (% tail DNA), was selected. The percentage tail DNA was measured by the intensity of the pixels located in the comet tail. Previous studies have shown the tail intensity parameter to be the most meaningful of the various comet parameters to assess genotoxicity [38] because the amount of DNA in the comet tail positively correlates with the level of DNA damage.

### 5.6. Statistical Analysis

Statistical analysis and graphical representations were performed using R version 4.1.0 [68] and R Studio packages *vegan* and *ggplot2*. Assumptions of normality (Shapiro Wilk test) and homoscedasticity (Levene’s test) for one-way analysis of variance (ANOVA) were met using log-transformed data for the reproduction and comet assay results. The analysis of controls between temperature and moisture treatments were conducted using two-way ANOVA. Further, three-way ANOVA was performed to test the possible interaction effect of concentration × moisture × temperature. Post hoc analysis (Tukey’s HSD) was performed following ANOVA. The MyCurveFit Add-in for Microsoft Excel was used for the aflatoxin concentration data to create a standard curve and perform a four-parameter logistics regression model (4PL) for concentration calculations from the optical density values.

## Figures and Tables

**Table 1:** Summary of the three-way ANOVA on the effect of aflatoxin concentration (C), moisture (WHC) and temperature on DNA damage and reproduction after 30 days of exposure, df-degrees of freedom. Statistically significant differences are indicated with asterisks. * *p* < 0.5; ** *p* < 0.01; *** *p* < 0.001.

	DNA Damage		Reproduction	
	df	F-Value	df	F-Value
Concentration (C)	2	17.616 ***	2	3.509 *
Moisture (WHC)	1	36.458 ***	1	80.222 ***
Tempertature (T)	1	131.683 ***	1	0.107
C × WCH	2	2.895 *	2	0.024
C × T	2	0.852	2	2.474
T × WHC	1	8.817 **	1	11.867 **
C × T × WHC	2	0.698	2	1.618

**Table 2:** Pairwise comparisons with Tukey’s HSD to determine the specific interaction effect of temperature (T) and moisture (WHC) during the comet assay (DNA damage) and reproduction test. Statistically significant (*p* < 0.05) interaction effects are indicated by asterisks. ** *p* < 0.01; *** *p* < 0.001.

Temperature (T) × Moisture (WHC)		
Temp_WHC	DNA Damage	Reproduction
21_50 × 21_30	***	**
26_30 × 21_30	***	
26_50 × 21_30	***	***
26_30 × 21_50	***	***
26_50 × 21_50	***	
26_50 × 26_30		***

**Table 3:** Aflatoxin concentrations detected in environmental soil samples.

	Aflatoxin Concentration	Environmental Sample Analysed	Detection Method
Mertz et al. [3]	0.1–10 µg/kg	Agricultural soil	TLC
Accinelli et al. [12]	0.6–5.5 µg/kg 145–275 µg/kg	Soil Decomposing corn residues	HPLC
Hariprasad et al. [4]	0.5–22 µg/kg	Soil samples	Indirect competitive (ic) ELISA
Rajkumar et al. [66]	10–100 µg/kg 50–1700 µg/kg	Soil samples Decomposing maise residues	LC-MS

## Data Availability

The data that support the findings presented in this study are available on request from the corresponding author.
